# Sleep Habits of Elementary and Middle School Children in South Texas

**DOI:** 10.1155/2015/179103

**Published:** 2015-12-03

**Authors:** Salim Surani, Sean Hesselbacher, Saherish Surani, Sreevidya Sadasiva, Zoya Surani, Sara S. Surani, Amina Khimani, Shyam Subramanian

**Affiliations:** ^1^Texas A&M University, Corpus Christi, TX 78405, USA; ^2^Pulmonary Associates, Corpus Christi, TX 78336, USA; ^3^Sentara Healthcare, Virginia Beach, VA 23454, USA; ^4^Eastern Virginia Medical School, Norfolk, VA 23507, USA; ^5^Harvard University, Boston, MA 02138, USA; ^6^University of California, Berkeley, CA 94720, USA; ^7^Mercy Health, Cincinnati, OH 45202, USA

## Abstract

*Background.* Sleep difficulties, including insufficient sleep and inadequate sleep hygiene, have been prevalent among children. Sleep deprivation can lead to poor grades, sleepiness, and moodiness. We undertook this study to assess the prevalence of sleep abnormalities among elementary and middle school students in South Texas and how the groups compare with one another.* Method.* After approval from the appropriate school district for a sleep education program, a baseline survey was taken of elementary and middle school students, using the Children's Sleep Habit Questionnaire-Sleep Self-Report Form, which assessed the domains of bedtime resistance, sleep onset delay, sleep anxiety, sleep duration, night awakening, and daytime sleepiness.* Results.* The survey was completed by 499 elementary and 1008 middle school children. Trouble sleeping was reported by 43% in elementary school, compared with 29% of middle school children. Fifty percent of middle school children did not like sleeping, compared with 26% in elementary school. Bedtime resistance, sleep onset delay, and nighttime awakening were more common among elementary school students. Daytime sleepiness was more common among the middle school children when compared to elementary school children.* Conclusions.* Sleep abnormalities are present in elementary school children with changes in sleep habits into middle school.

## 1. Introduction

Insufficient sleep syndrome “occurs when an individual persistently fails to obtain the amount of sleep required to maintain normal levels of alertness and wakefulness. The individual is chronically sleep deprived as a result of failure to achieve necessary sleep time due to reduced time in bed.” Inadequate sleep hygiene is characterized by sleep and wake difficulties resulting from “daily living activities that are inconsistent with the maintenance of good-quality sleep and normal daytime alertness” [1]. These problems are common among adolescents and children [2–7].

The Centers for Disease Control currently recommend 9-10 hours of sleep nightly in teenagers and 10+ hours in school age children [8]; the National Sleep Foundation Scientific Advisory Council similarly recommends 9–11 hours for school age children and 8–10 hours for teenagers [9]. Only 15% of teens slept 8.5 hours or more on school nights [10] and 70% have a bedtime of 10 PM or later. Common reasons given for delayed sleep included homework, hanging out with friends, television, stress, and part-time jobs [5]. A recently published yearly cross-sectional survey of adolescents revealed that the percentage of teens achieving ≥7 hours of sleep nightly decreases each year from ages 12 through 18 and has shown an overall decline over the past 20 years [7]. Teens are more inclined to have irregular sleep patterns across the week and tend to stay up late and sleep in late on weekends [10]. Irregular sleep patterns are detrimental to the biological clock and affect sleep quality. Sleep deprivation can adversely affect academics, behaviors, and overall health [3, 4, 6, 11, 12].

Approximately, 90% of parents feel that their children are getting adequate sleep. Conversely, 60% of the adolescents reported difficulty getting out of bed in the morning and majority of them required their parents to wake them up for school [11, 13, 14]. These conflicting reports highlight discrepancies in the ways parents and children view the adequacy of the children's sleep.

We undertook this study to assess the prevalence of sleep-related problems among school children in South Texas, including inadequate sleep hygiene and insufficient sleep, and to ascertain some of the differences in sleep habits between children of elementary and middle school ages. The majority of the sleep related studies that have been conducted in United States of America and Europe have been on Caucasian population. Very little research has been done among US Hispanic population. Based on the demographic and prevalent risk factors as diabetes, obesity, low socioeconomic factors, high incidence of obstructive sleep apnea, and low educational status points toward sleep habit [15]. This is the first attempt to get the epidemiological information as it relates to sleep among children in South Texas. 65% of the population in the region is of Hispanic ethnicity, with low percentage of professionals, and only 21% of them are college graduates. There are 38,938 students enrolled in this school district; of those 78.9% are of Hispanic ethnicity [16]. Enrollment in the middle schools is larger than that of the elementary schools as several feeder elementary schools feed into one middle school; therefore, data were collected from 1 middle school and 2 elementary schools. We attempted to approximate the overall population demographics in the groups chosen to answer the questionnaires: the ethnic composition of the middle school is similar to that of the school district as a whole. Elementary school #1 has a higher Hispanic population, lower proportion of Caucasians, and lower socioeconomic status than the composition of the school district; the inverse is true of elementary school #2. We aimed to compare these groups based on self-reported sleep habits, which can often be different from the perception of the parents.

## 2. Methods

Participants were elementary (4th and 5th grades) and middle (6th through 8th grades) school students taking part in a school-sponsored sleep education program. Permission was obtained from the Corpus Christi Independent School District prior to embarking on the broader sleep education program. All elementary and middle schools in the school district were offered participation in the sleep education program; 2 elementary and 1 middle schools were chosen to participate in the first wave of education, with the baseline data reported here being collected from these schools. The schools were felt, by consensus of the investigators, to represent the overall school district as closely as possible. All participants completed the Children's Sleep Habits Questionnaire-Sleep Self Report Form (CSHQ-SSRF). The CSHQ is a validated tool, assessing students in the domains of bedtime resistance, sleep onset delay, sleep anxiety, sleep duration, night awakening, and daytime sleepiness, which has demonstrated good internal consistency in both community and clinical samples [17]. The CSHQ-SSRH is a 26-item survey, which is designed to assess sleep domains similar to the CSHQ, completed by the student (rather than parent or caregiver) [18, 19]. The items are grouped into six blocks, with each addressing an aspect of sleep quality/quantity, and are answered based on a 3-point scale. The points system designates “usually” (5–7 times/week), “sometimes” (2–4 times/week), and “rarely” (0-1 times/week); a higher score indicates more abnormal sleep [20]. The sum of the scores for the whole questionnaire (questions 2 through 26) has a possible maximum score of 73 and a minimum score of 23. All completed questionnaires were accepted for analysis; incomplete questionnaires were excluded.

### 2.1. Statistics

**Figure 1:**
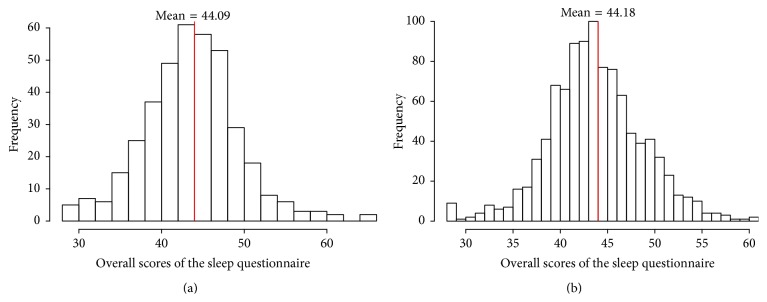
Distribution of the total questionnaire scores for elementary (a) and middle (b) school students.

Statistical analysis was performed using R Statistical Software (Foundation for Statistical Computing, Vienna, Austria). The proportion of responses to the questionnaire was calculated to determine baseline sleep habits and problems. The mean scores were calculated for each question and the overall questionnaire. *t*-test was performed on the mean scores of each group of questions (except group 2 which is a single question) to detect the differences between the groups. As shown in Figure 1, the distribution of total questionnaire scores for each group of students was normally distributed; the same was assumed to hold true for groups of multiple questions. Because the responses to single questions were categorical data, the proportion of responses to each question was compared between the groups using contingency tables. A *P* value of <0.05 was considered statistically significant.

## 3. Results

### 3.1. Demographic Characteristics

A total of 499 elementary school students (360 4th grade, 139 5th grade) submitted questionnaires, of which 387 were complete; 1000 of 1008 middle school surveys submitted were complete. Demographic data of the individual respondents are unavailable. The demographic characteristics of the students in each school in the most recent year for which data are available (2010) are represented in Table 1. All schools had a predominance of Hispanic ethnicity. The ethnic make-up of elementary school #2 (ES2) was significantly different than that of elementary school #1 (ES1, *P* < 0.0001) and the middle school (MS, *P* < 0.0001), which were similar to each other. The population of ES2 was comprised of fewer Hispanics and African Americans and more Caucasians and Asians/Pacific Islanders than the other 2 schools. A significantly higher proportion of students in ES1 were economically disadvantaged than in MS, which had a higher proportion than ES2; similar findings were identified for at-risk children. Both elementary schools had significantly more students with limited English proficiency than the MS, though they were similar to each other in this respect.

### 3.2. Sleep Habits of Elementary School Students

The questionnaire total scores ranged from 28 to 65 (of a possible 73), with a mean of 44 as can be seen in Figure 1(a). Responses to the individual questions are listed in Table 2. Of the 387 students that completed the questionnaire, 43% had trouble sleeping and 26% did not like to sleep. Major sleep issues were seen in different blocks of the questionnaire in 10%–40% of students: 44% of the students rarely slept in the same bed; 31% usually stayed up late when parents thought they were asleep; only 28% of students fell asleep within 20 minutes; 34% rarely felt rested after a good night's sleep.

### 3.3. Sleep Habits of Middle School Students

Among the middle school students, the total questionnaire scores ranged from 28 to 61 with mean of 44 which is graphically depicted in Figure 1(b). The breakdown of the responses to each question is detailed in Table 3: 29% had trouble sleeping and 50% did not like to sleep; 10%–50% of students had major sleep issues in different blocks of the questionnaire; 50% of the students rarely sleep alone; 50% are rarely ready to go to bed at usual bedtime; approximately one-third of students usually find it difficult to go to bed; 40% take naps during day time; only 22% of students fell asleep within 20 minutes; 40% felt rested after a good night's sleep.

### 3.4. Comparison of Elementary and Middle School Data

A question-by-question comparison between elementary and middle school students is detailed in Table 4. There was a significant difference in the percent of students who had trouble sleeping and 43% in elementary compared to 29% in middle school (*P* < 0.01). Also, the percentage of students who did not like sleeping was different between the groups, 26% in elementary and 50% in middle school (*P* < 0.01). The two groups differed significantly on 20 of the 25 questions by Chi square analysis.

Responses to the Bedtime Resistance block of questions differed significantly between the 2 groups (*P* = 0.01). Middle school students were more likely to go to bed at the same time (*P* < 0.01) on school nights, in the same bed (*P* < 0.01), and were less likely to stay up late when the parents thought they were asleep (*P* < 0.01). In the same block of questions, elementary students answered that they fought over going to bed less frequently (*P* < 0.01) and were more often ready to go to bed at the usual bedtime (*P* = 0.01). Only 22% of middle school students reported falling asleep within 20 minutes, compared with 36% of elementary school students (*P* < 0.01). The Sleep Anxiety block of questions did not differ overall. Elementary students more often reported taking a special thing to bed (*P* < 0.01) and being afraid of the dark (*P* < 0.01) but were less often afraid of sleeping alone (*P* < 0.01). More elementary students answered that they sleep too little (*P* < 0.01) and too much (*P* < 0.01), though the overall Sleep Duration block did not demonstrate a significant difference. The Night Awakening block of questions was not different between the groups. More elementary students reported nightmares (*P* < 0.01) and having trouble falling back asleep after awakening (*P* < 0.01), though middle school students more often go to someone else's bed during the night (*P* < 0.01). Middle schools students were more sleepy than elementary students overall (*P* < 0.01), more often reporting feeling sleepy during the day (*P* < 0.01), taking naps (*P* < 0.01), and feeling rested after a night's sleep (*P* < 0.01). The total questionnaire scores were not statistically significant (*P* = 0.78).

## 4. Discussion

Our study has demonstrated the presence of sleep problems in a significant proportion of elementary and middle school children in a predominantly Hispanic population in South Texas; there are several important differences in the self-reported sleep habits between these two groups. Elementary students reported both trouble sleeping and liking sleeping more often than middle school students. Notable differences between the two groups included bedtime resistance, sleep onset delay, and daytime sleepiness. The total questionnaire scores were similar in both groups of students, underscoring the importance of individual and grouped question responses in addition to the composite score.

Assessing adequacy of sleep hygiene in these populations was a major aim of this study. Middle school students appeared to adhere to traditional measures of sleep hygiene, such as consistent bedtime and location, compared to the elementary students; however, they reported more disobedient behaviors, such as fighting with their parents over going to bed. Elementary students more often stayed up after their parents thought they were asleep, though they were more likely to report falling asleep within 20 minutes. Our students were less likely to fall asleep within 20 minutes than those surveyed by a National Sleep Foundation (NSF) poll in 2004, in which 22% responded that they take more than 20 minutes to fall asleep [21]. Electronics and other toys in the bedroom can be used to discretely violate household rules; small screens (e.g., smartphones) and televisions in the bedroom have recently been associated with shorter sleep duration in a survey of 4th through 7th graders [22]. Both of our groups felt that they got an inappropriate amount (too much or too little) of sleep at most once a week. The sleep resistance answers obtained here were similar to another self-reported survey that recognized bedtime resistance in 27% of elementary school students [2]; this was by far the most common sleep problem identified in that group. The 2004 NSF poll reported that 42% children stall in going to bed [21].

Sleep anxiety has complicated interactions with sleep and may contribute to some of the sleep difficulties, potentially explaining aberrations in sleep hygiene. Middle school students were significantly more likely to report fear of sleeping alone; they also more often sleep with someone else and go to someone else's bed during the night. Conversely, elementary students were more afraid of the dark and had nightmares and trouble falling back to sleep after awakening. This study was not designed to elucidate the reasons behind these fears, or whether the fears motivated the behaviors that were associated.

Causes of daytime sleepiness are multifactorial and may include intrinsic sleep disorders such as sleep-disordered breathing or narcolepsy, insufficient sleep syndrome, medical comorbidities, or medications. Self-identification of daytime sleepiness can be unreliable in children and adults. Questionnaires have been developed for this purpose, often asking the subject about falling asleep or dozing off in certain situations; some of these questionnaires include the Epworth Sleepiness Scale [23, 24] and the Children's Report of Sleep Patterns-Sleepiness Scale [25]. Another example is seen in this study: more than 40% of middle school students reported taking naps during the day 5–7 times per week, while only 23% reported feeling sleepy during the day on a usual basis. The proportion of these children that reported taking naps was unexpected, given that all are >5 years of age. However, these results are similar to an earlier study that showed that 32% of a group of 9–11-year-old children took daytime naps [26]. In that study, nappers did not report differences in nighttime sleep (though actigraphy showed shorted nighttime sleep) or more daytime sleepiness than nonnappers. Almost 30% of all students surveyed reported difficulty waking up 5–7 days per week (similar in both groups). The previously mentioned NSF poll found that 29% of children under age 10 had difficulty waking up at least a few mornings per week [21]; the self-report survey done by Blader and associates showed that 17% had morning wake up problems [2].

The CSHQ was initially designed to be completed by a parent or caregiver; we used a modified version designed to be answered directly by the child. We felt it was important to obtain the answers from the children directly for a number of reasons. First, children may become more independent and/or learn to hide certain “wrong” behaviors from their parents with age [11]; thus, parent-reported answers might introduce bias into some of the comparisons of interest. Both sets of respondents may be susceptible to social desirability bias, albeit from different sources. A parent may skew the answers to reflect “proper” sleep habits that have been disbursed by the media, peers, or their parents over many years. A child may similarly skew his or her answers, though the information is more limited, and the rules of the house can be presumed to play a larger role in the sense of right and wrong; thus, while the answers would still deviate from the true sleep habits, they would reflect a point of emphasis in the household. Previous studies have validated self-reported sleep habits from similar aged children [25, 27, 28] and in adolescents [13]. Children self-report identified more sleep problems than parent report in a comparative study [20], though parent-reported mean scores on the parent-answered CSHQ from a community sample (56.2) [17] was higher than the mean scores for the student-answered surveys (44 in both groups) in the present study. No standardized assessment measures exist for testing sleep knowledge in children. Because of this, investigators construct and develop their own questionnaires for use in research settings; this limits clinical application. Blunden and associates have highlighted the need for such standard sleep knowledge measures [29]. The questionnaire we used in this study attempts to address the problem of insufficient sleep in children but does not directly quantify the amount of sleep achieved (or attempted). The questions ask the child to answer whether they think they get too much or too little sleep. Properly answering this question would require baseline knowledge of the appropriate amount of sleep for their age, which was also not assessed in this study.

An important consideration of our study includes the demographic breakdown of our study sample. The schools participating in this survey were predominantly Hispanic, reflecting the make-up of the community at large. Ethnicity and gender are known to have significant effects on sleep behaviors. Non-Caucasian children reported later bedtimes and had shorter sleep duration than Caucasian children [30–33]. Previous studies have reported that boys had shorter sleep duration than girls on nonschool nights [30, 32–34]. An actigraphic study found that girls and nonminority adolescents achieved more sleep [35] than others. Another study conducted on minority children found that girls aged 11-12 years slept 0.3 hours less than boys per day [36]. A much higher percentage of Hispanic children (74%) have televisions in their bedroom, compared with white children (22%) [37], which has a detrimental association with sleep duration [22]. As noted previously, much of the prior research on pediatric sleep habits has been done on primarily Caucasian populations; this difference may explain some of the discrepancies noted between our results and prior reports, including the NSF poll [20]. Ethnic and/or gender factors may affect how the answers from a parent and child might differ with respect to the child's sleep, as in this report. Collection of gender, age, and ethnicity data for the individual students was not possible within the constructs of this study. Therefore, any conclusions regarding the impact of each of these factors would be speculative. Participation in this questionnaire was done in large groups and classes; thus, it is felt that the participating student population is representative of the overall population of each school. These results cannot be assumed to pertain to populations other than the group described here. Replication of these results in other ethnic and socioeconomic groups may allow these findings to confidently be applied to a broader population.

Similarly, the lack of individual age data is a significant limitation. Texas state law requires that children between the ages of 6 and 18 years attend school; those 6 years of age on September 1 of that year are enrolled in first grade and they progress 1 grade level each September, provided acceptable academic and social progress. While children in a given school grade level are typically of a similar age, this assumption does not account for children that have been held back or “skipped” a grade for any reason or transferred from another education system with different requirements. Importantly, sleep habits are not the product of age alone; social interactions (friends, family) play a central role in behaviors, including sleep habits. While these results should not be applied to children of specific ages due to these limitations, they may be viewed in the context of social maturation and academic progression through school.

Because of the large number of questions in the questionnaire, multiple comparisons were performed, which introduces concern for Type 1 error. Because of the large number of data sets used and because of the high proportion of questions with significant differences (20/25 individual questions), the risk of such an error meaningfully altering the results is very low, so additional statistical adjustments were not performed.

Interventions for children and adolescents have been developed to address many of the sleep problems identified here through educations programs. The Australian Centre for Education in Sleep (ACES) conducted sleep education programs in Australia and New Zealand, using a PowerPoint lecture to increase sleep knowledge and improve sleep behaviors in adolescents. Significant improvements on sleep knowledge were shown in the Australian trials, and the New Zealand trial showed significant improvements in week and weekend sleep duration [38]. Positive feedback regarding the program was received from both teachers and students involved in these trials. Another unrelated sleep education study was performed in Australia [39]. This intervention consisted of four 50-minute classes over 4 weeks; while sleep knowledge improved, the target sleep variables did not. Our data show that disrupted sleep appears to begin younger than adolescence, so earlier intervention may be warranted. The current survey was part of a larger educational program, known as Keep Nurture and Inspire Good Habits in Teen Sleep (KNIGHTS), which utilizes a 3D animated sleep education movie to promote healthy sleep habits in elementary and middle school students [40].

## 5. Conclusion

Sleep abnormalities are present by the time children are in 4th and 5th grades, with changes as they progress into middle school. Middle school students reported more adherence to traditional measures of sleep hygiene than the elementary students; however, they reported more disobedient behaviors. Bedtime resistance, sleep onset delay, and nighttime awakenings are more common among elementary school students, though middle school students are more likely to argue with their parents about going to bed. These differences may be attributed to a number of factors, including neurobiological development, evolving social awareness and interactions, and intrusion of generalized behavioral changes into sleep. Self-reported daytime sleepiness is more prevalent among middle school students. Educational efforts may be useful in guiding proper sleep habits if started at a younger age; this warrants further study. Further studies are suggested to look at specific age group and gender to help identify these issues even better.

## Figures and Tables

**Table 1:** Demographic characteristics of the students.

School	Elementary #1	Elementary #2	Middle
Ethnicity			
Hispanic	68.5%	47.4%	67.3%
Caucasian	24.4%	39.8%	23.6%
African American	5.8%	2%	6%
Asian/Pacific Islander	1.3%	10.1%	2.9%
Native American	0%	0.6%	0.2%
Risk factors			
Economically disadvantaged	58.7%	29.4%	36.8%
At risk	42.3%	18.3%	30%
Limited English proficient	1.9%	3.8%	0.1%
Teacher experience (mean years)	16.8	17.6	15.8

**Table 2:** Responses to the Children's Sleep Habits Questionnaire from elementary school students.

Qn #		Yes	No	
2	Trouble sleeping	43.04%	56.96%	
3	Likes to sleep	73.55%	26.45%	

		Rarely	Sometimes	Usually

*Block 1: bedtime resistance*				
4	Goes to bed at same time	30.16%	36.64%	33.20%
5	Falls asleep in same bed	32.25%	27.99%	39.76%
6	Sleeps alone	37.96%	24.69%	37.35%
7	Falls asleep in other's bed	46.41%	30.80%	22.79%
9	Fights with parents to go to bed	61.20%	25.93%	12.86%
10	Hard to go to bed	41.36%	33.33%	25.31%
11	Ready to sleep at usual bedtime	43.67%	30.00%	26.33%
15	Stay up late when parents think you are asleep	39.51%	28.72%	31.77%

*Block 2: sleep onset delay*				
8	Falls asleep in 20 minutes	36.21%	36.01%	27.78%

*Block 3: sleep anxiety*				
12	Takes a special thing to bed	48.05%	25.05%	26.90%
13	Afraid of dark	60.33%	20.45%	19.22%
14	Afraid of sleeping alone	58.80%	25.47%	15.73%

*Block 4: sleep duration*				
16	Sleeps too little	48.98%	28.69%	22.34%
17	Sleeps too much	55.56%	28.19%	16.26%

*Block 5: night awakening*				
18	Wakes up at night when parents think you are asleep	47.20%	30.23%	22.57%
19	Trouble falling back asleep after waking up	45.74%	28.90%	25.36%
20	Have nightmares	48.35%	33.13%	18.52%
21	Wakes up due to pain	57.02%	27.04%	15.93%
22	Goes to other's bed during night	60.96%	28.39%	10.65%

*Block 6: daytime sleepiness*				
23	Trouble waking up in morning	37.22%	35.17%	27.61%
24	Feels sleepy during day	41.31%	40.29%	18.40%
25	Takes naps during day	56.03%	28.63%	15.34%
26	Feels rested after a night's sleep	33.61%	30.53%	35.86%

Qn #: question number.

**Table 3:** Responses to the Children's Sleep Habits Questionnaire from middle school students.

Qn #		Yes	No	
2	Trouble sleeping	28.77%	71.23%	
3	Likes to sleep	50.00%	50.00%	

		Rarely	Sometimes	Usually

*Block 1: bedtime resistance*				
4	Goes to bed at same time	11.21%	30.06%	58.73%
5	Falls asleep in same bed	26.49%	20.73%	52.78%
6	Sleeps alone	50.99%	23.61%	25.40%
7	Falls asleep in other's bed	48.81%	30.56%	20.63%
9	Fights with parents to go to bed	50.79%	27.08%	22.12%
10	Hard to go to bed	39.48%	31.65%	28.87%
11	Ready to sleep at usual bedtime	50.89%	23.21%	25.89%
15	Stay up late when parents think you are asleep	47.92%	33.13%	18.95%

*Block 2: sleep onset delay*				
8	Falls asleep in 20 minutes	47.67%	30.09%	22.24%

*Block 3: sleep anxiety*				
12	Takes a special thing to bed	58.33%	20.63%	21.03%
13	Afraid of dark	58.13%	28.57%	13.29%
14	Afraid of sleeping alone	43.15%	33.93%	22.92%

*Block 4: sleep duration*				
16	Sleeps too little	45.46%	37.49%	17.05%
17	Sleeps too much	44.47%	39.98%	15.55%

*Block 5: night awakening*				
18	Wakes up at night when parents think you are asleep	41.27%	36.01%	22.72%
19	Trouble falling back asleep after waking up	52.98%	30.06%	16.96%
20	Have nightmares	61.71%	23.91%	14.38%
21	Wakes up due to pain	61.90%	22.22%	15.87%
22	Goes to other's bed during night	43.55%	27.78%	28.67%

*Block 6: daytime sleepiness*				
23	Trouble waking up in morning	41.35%	31.81%	26.84%
24	Feels sleepy during day	47.71%	28.83%	23.46%
25	Takes naps during day	29.92%	29.42%	40.66%
26	Feels rested after a night's sleep	23.96%	36.78%	39.26%

Qn #: question number.

**Table 4:** Comparisons of sleep habits between elementary and middle school students.

Qn #	Item description	*P* value (*t*-test)	*P* value (*χ* ^2^)
2	Trouble sleeping		0.00^*∗*^
3	Likes to sleep		0.00^*∗*^
*Block 1: bedtime resistance*		0.01^*∗*^	
4	Do you go to bed at same time every night on school nights?		0.00^*∗*^
5	Do you fall asleep in same bed every night?		0.00^*∗*^
6	Do you fall asleep alone?		0.00^*∗*^
7	Do you falls asleep in parents', brother's, or sister's bed?		0.57
9	Do you fight with your parents about going to bed?		0.00^*∗*^
10	Is it hard for you to go to bed?		0.35
11	Are you ready for bed at your usual bedtime?		0.01^*∗*^
15	Do you stay up late when parents think you are asleep?		0.00^*∗*^
*Block 2: sleep onset delay*			
8	Do you fall asleep in about 20 minutes?		0.00^*∗*^
*Block 3: sleep anxiety*		0.82	
12	Do you have a special thing you bring to bed?		0.00^*∗*^
13	Are you afraid of the dark?		0.00^*∗*^
14	Are you afraid of sleeping alone?		0.00^*∗*^
*Block 4: sleep duration*		0.15	
16	Do you think you sleep too little?		0.00^*∗*^
17	Do you think you sleep too much?		0.00^*∗*^
*Block 5: night awakening*		0.66	
18	Do you wake up at night when parents think you are asleep?		0.05
19	Do you have trouble falling back to sleep if you wake up during the night?		0.00^*∗*^
20	Do you have nightmares?		0.00^*∗*^
21	Does pain wake you up at night? Where is that pain?		0.11
22	Do you sometimes go to someone's bed during the night? If yes who?		0.00^*∗*^
*Block 6: daytime sleepiness*		0.00^*∗*^	
23	Do you have trouble waking up in the morning?		0.27
24	Do you feel sleepy during the day?		0.00^*∗*^
25	Do you take naps during the day?		0.00^*∗*^
26	Do you feel rested after a night's sleep?		0.00^*∗*^
*Questions 2 to 26 (overall score)*		0.78	

^*∗*^ *P* < 0.05.

Qn #: question number; *χ* ^2^: chi square.
